# Quality of life after robot-assisted transmediastinal radical surgery for esophageal cancer

**DOI:** 10.1007/s00464-017-5918-x

**Published:** 2018-03-01

**Authors:** Shuntaro Yoshimura, Kazuhiko Mori, Yukinori Yamagata, Susumu Aikou, Koichi Yagi, Masato Nishida, Hiroharu Yamashita, Sachiyo Nomura, Yasuyuki Seto

**Affiliations:** 1 Department of Gastrointestinal Surgery, Graduate School of Medicine, University of Tokyo, 7-3-1 Hongo, Bunkyo-ku, Tokyo, 113-8655 Japan; 2 Department of Gastrointestinal Surgery, Mitsui Memorial Hospital, 1 Kanda Izumi, Chiyoda, Tokyo, 101-8643 Japan; 3 Department of Surgery, Dokkyo Medical University Koshigaya Hospital, 2-1-50 Minamikoshigaya, Koshigaya, Saitama, 343-8555 Japan

**Keywords:** Esophagus cancer, Esophagectomy, Minimally invasive esophagectomy, Robot-assisted esophagectomy, Quality of life

## Abstract

**Background:**

The aim of this retrospective study was to assess postoperative quality of life (QOL) after robot-assisted radical transmediastinal esophagectomy, defined as a nontransthoracic esophagectomy with radical mediastinal lymphadenectomy combining a robotic transhiatal approach and a video-assisted cervical approach. The results were compared to those of transthoracic esophagectomy.

**Methods:**

In this study, all consecutive patients who underwent robot-assisted radical transmediastinal esophagectomy or transthoracic esophagectomy for esophageal cancer at University of Tokyo between January 2010 and December 2014 were included. The European Organization for Research and Treatment of Cancer (EORTC)’s quality of life questionnaires QLQ-C30 and QLQ-OES18 were sent to all patients that were still living, had no recurrence or other malignancy, and had not undergone a reoperation because of complications after esophagectomy.

**Results:**

We were able to survey 63 (98.4%) of 64 eligible patients. We assessed and compared the QOL scores of both groups of patients. Compared to transthoracic esophagectomy, transmediastinal esophagectomy was associated with better QOL. Global health status and the physical, role, and cognitive function scale scores were significantly superior in the transmediastinal esophagectomy group (*P* = 0.004, < 0.0001, 0.007, 0.002, respectively). Fatigue, nausea and vomiting, pain, appetite loss, reflux, and taste scores were significant lower (superior) in the transmediastinal esophagectomy group (*P* = 0.003, 0.032, 0.025, 0.018, 0.001, 0.041, respectively).

**Conclusions:**

This study indicates that robot-assisted radical transmediastinal esophagectomy is associated with better postoperative QOL compared to transthoracic esophagectomy. A larger study and prospective analyses are needed to confirm the current results.

Radical esophagectomy for esophageal cancer is still a challenging surgery because of its high morbidity and mortality [1]. Whereas perioperative mortality rates have fallen to 3.4%, postoperative morbidity rates remain high, at approximately 40% [2]. Progress has been made in minimally invasive esophagectomy for esophageal cancer [3] and a randomized control trial has shown evidence for the short-term benefits of video-assisted thoracoscopic esophagectomy compared with open esophagectomy [4]. However, a recent report on the Japanese national database revealed that minimally invasive esophagectomy increases the rate of surgical complications [2]. Further efforts should be made to reduce the morbidity and mortality of esophagectomy. Transhiatal esophagectomy is also a favored choice, with lower perioperative morbidity, but the oncological outcome of the transhiatal approach is generally considered inferior, since only limited lymph nodes can be harvested when compared to the transthoracic approach [5].

To overcome these shortcomings, we developed transmediastinal esophagectomy, a nontransthoracic esophagectomy with radical mediastinal lymphadenectomy combining a robotic transhiatal approach and a video-assisted cervical approach reported previously by the authors [6]. In our previous study, which compared transmediastinal esophagectomy with conventional transthoracic esophagectomy, we found that postoperative hospital stays were shorter and postoperative pneumonia did not occur in the transmediastinal esophagectomy group [7]. The same study also demonstrated that the radicality of transmediastinal esophagectomy was equivalent to that of transthoracic esophagectomy in terms of the number of harvested lymph nodes and surgical margin pathology [7].

Patients’ quality of life (QOL) after transmediastinal esophagectomy accompanying radical mediastinal dissection was still unknown, however. The object of the present study was to assess postoperative QOL after transmediastinal esophagectomy.

## Materials and methods

### Patients

Esophageal cancer patients who underwent transmediastinal esophagectomy or transthoracic esophagectomy with gastric conduit reconstruction via the posterior mediastinal route between January 2010 and December 2014 at the University of Tokyo Hospital were candidates for this study. All these patients were staged preoperatively using esophagogastroduodenoscopy with biopsies and computed tomography scans. Transmediastinal esophagectomy was performed using a robotic surgical system as described in our previous clinical study verifying the safety and utility of robotic transmediastinal esophagectomy [7]. The indications for transthoracic or transmediastinal esophagectomy are (1) histologically proven esophageal cancer, (2) sufficiently good general condition to tolerate conventional open esophagectomy, and (3) a tumor clinically staged as T1-3 N0-1 M0 according to the 7th edition of the American Joint Committee on Cancer tumor-node-metastasis (TNM) classification. In addition, written informed consent to undergo robot-assisted surgery without receiving financial support from the national health insurance system was required for transmediastinal esophagectomy. The QOL survey was performed after excluding those of the above-described patients who met the following exclusion criteria: (1) patients who had a recurrent lesion or who were under treatment for other malignancies; (2) patients who had a history of surgeries of another malignancy; (3) patients who had undergone a reoperation because of complications after esophagectomy. Between January 2010 and December 2014, 128 esophageal cancer patients underwent transthoracic esophagectomy or transmediastinal esophagectomy. Of the 128 patients, 17 patients had died before this study began, 18 patients developed disease recurrence, and 2 patients had a history of reoperation for postoperative complications after esophagectomy. According to the exclusion criteria, consequently, 64 patients, 26 transmediastinal esophagectomy patients, and 38 transthoracic esophagectomy patients, were subjects in the present study, in which we assessed the QOL scores of post-esophagectomy patients and compared the transmediastinal esophagectomy and the transthoracic esophagectomy group scores. This study was approved by The University of Tokyo’s institutional review board. All study participants provided informed consent, and all 64 patients gave their consent.

### Surgical methods

The robot-assisted transmediastinal esophagectomy with three-field lymphadenectomy was performed in three stages, all with the patient in the supine position. In the first stage, lymph node dissections in the cervical and the abdominal fields were performed simultaneously by two surgical teams. The cervical procedure was performed via a collar incision under mediastinoscopic guidance. The abdominal procedure was performed via a laparoscopic approach. In the second stage, the robotic surgical device, da Vinci S (Intuitive Surgical, Sunnyvale, CA, USA), was brought in to perform the transhiatal robotic procedure through the abdominal ports. In the dissections consisting of the cervical procedure via the collar incision and the da Vinci procedure via the transhiatal approach, the entire esophagus as well as dissected mediastinal lymph nodes was freed from adhesions and attachments. Upon completion of the mediastinal dissection, the da Vinci S robotic system was moved away from the surgical field. The last stage included the harvest of surgical specimens, reconstruction with a gastric tube conduit, and cervical anastomosis.

Transthoracic esophagectomy patients underwent a right anterolateral thoracotomy via the fourth intercostal space with two- or three-field lymphadenectomy and intrathoracic anastomosis. The creation of the gastric conduit was performed by the same procedure as that performed in the transmediastinal esophagectomy: a gastric conduit with a diameter of 4 cm was created with linear staplers. Pyloroplasty was performed by the same procedure in both transthoracic esophagectomy and transmediastinal esophagectomy. The posterior mediastinal route was used with only one exception, and the anastomosis was performed using a 25-mm circular stapler.

### Quality of life measurement

The cross-sectional QOL survey evaluated patients at intervals of more than 3 months since their esophagectomy or the last administration of chemotherapeutic agents from October 2014 to September 2015. Patients who met the above criteria were asked to participate in this study on their regular follow-up visit. The participants were given a self-administered questionnaire and were asked to send it back by mail after filling out the queries.

Patients’ quality of life was assessed using the validated European Organization for Research and Treatment of Cancer Quality of Life Core Questionnaire (EORTC QLQ-C30) [8] as well as the esophageal site-specific module (EORTC QLQ-OES18) [9]. The EORTC QLQ-C30 consists of the patient’s global health status, five functional scales (physical, role, emotional, cognitive, and social functioning), three symptom scales (fatigue, pain, and nausea and vomiting) and six single-item measures (dyspnea, insomnia, appetite loss, constipation, diarrhea, financial difficulties). It can assess functional aspects of QOL and symptoms that commonly occur in patients with cancer. The EORTC QLQ-OES18 consists of nine symptom scales (eating, reflux, pain, trouble swallowing saliva, choking when swallowing, dry mouth, trouble with taste, trouble with coughing, and trouble speaking), and was designed to assess patients treated for esophageal cancer by procedures including esophagectomy, chemoradiation, endoscopic palliation, or palliative chemotherapy and/or radiotherapy. High scores in the global health status and the function scales represent a higher level of function and enhanced global health status. On the other hand, higher scores in symptom scales represent more severe symptoms. The reliability and validity of the Japanese version of the EORTC QLQ-C30 and OES-18 have been demonstrated [10, 11].

### Statistical analysis

All statistical analyses were performed using JMP 11.0 (SAS Institute Inc. Cary, NC, USA). Wilcoxon’s rank-sum test was used for the analysis of group differences and Fisher’s exact test was used for the proportional differences. A *P* value less than 0.05 was considered statistically significant.

## Results

Esophageal cancer recurred in 14 (15.4%) of the 91 patients in the transthoracic group and in 4 (10.8%) of the 37 patients in the transmediastinal group; there was no significant difference (*P* = 0.59). Each of the two groups had one patient who had a history of reoperation; there was no significant difference (*P* = 0.50). There was, however, a significant difference in the current study population in terms of mortality: 16 cases (17.6%) among the 91 patients in the transthoracic group, 1 case (2.7%) among the 37 patients in the transmediastinal group (*P* = 0.023).

Of the 64 patients meeting the criteria for assessment of QOL, we were able to survey 63 patients (98.4%) using EORTC QLQ-C30 and QLQ-OES18. The patients’ characteristics are shown in Table 1. There were significant differences in clinical and pathological tumor stage between the two groups (*P* = 0.005, 0.043, respectively). However, there were no significant differences in clinical and pathological lymph node stage, nor were there significant differences in pathological stage between the two groups. Postoperative complications rated greater than Grade 2 by the Clavien–Dindo Classification [12] are shown in Table 2. The diagnosis of postoperative pneumonia was made in accordance with the Japanese Respiratory Society’s Guidelines for Hospital Acquired Pneumonia in Adults [13]. Pneumonia was significantly more frequent (*P* = 0.008) in the transthoracic group (24.3%) than in the transmediastinal group (0%). Anastomotic leakage was more frequent (though not significantly) (*P* = 0.707) in the transmediastinal (15.4%) than in the transthoracic group (10.8%). The median time point of assessment from esophagectomy was 23 months (range 6–37) in the transmediastinal group and 24 months (range 3–59) in the transthoracic group (no significant difference).

**Table 1:** Clinicopathological characteristics

	NTTE (*n* = 26)	TTE (*n* = 37)	*P* value^a^
Median age (range)	66 (48–82)	69 (46–92)	0.121
Gender (M/F)	24/2	30/7	0.236
Median BMI (range)	22.5 (16.8–29.4)	21.6 (17.4–29.5)	0.148
Brinkman Index (0/1-600/601–1200/1201-)	4/9/12/1	11/13/9/4	0.250
Location (Proximal/middle/distal/EGJ)	4/11/10/1	1/13/20/3	0.226
Clinical classification			
cT status (1/2/3)	16/6/4	9/9/19	0.005^a^
cN status (0/1)	14/12	24/13	0.44
Number of three-field lymphadenectomies (%)	17 (65.4%)	16 (43.2%)	0.124
Pathological classification			
pT status (1/2/3)	19/2/5	15/5/17	0.043^a^
pN status (0/1/2)	14/10/2	20/8/9	0.164
pStage (IA/IB/IIA/IIB/IIIA/IIIB)	4/7/2/9/3/1	4/6/6/7/11/3	0.301
Histological type (SCC/AC/other)	26/0/0	30/4/3	0.061

	Number of cases (%)		*P* value^a^
Neoadjuvant chemotherapy: *n* (%)	0 (0%)	1 (2.7%)	1.00
Adjuvant chemotherapy: *n* (%)	10 (38.5%)	12 (32.4%)	0.302

*AC* adenocarcinoma, *BMI* body mass index, *EGJ* esophagogastric junction, *NTTE* nontransthoracic esophagectomy, *SCC* squamous cell carcinoma, *TTE* transthoracic esophagectomy

^a^Fisher’s exact test

**Table 2:** Postoperative complications

	NTTE (*n* = 26)	TTE (*n* = 37)	*P* value^a^
	Number of cases (%)		
Pneumonia	0 (0%)	9 (24.3%)	0.008^a^
Anastomotic leakage	4 (15.4%)	4 (10.8%)	0.707
RLN palsy	2 (7.7%)	3 (8.1%)	1.00
Chylothorax	1 (3.9%)	2 (5.4%)	1.00

*NTTE* nontransthoracic esophagectomy, *RLN* recurrent laryngeal nerve, *TTE* transthoracic esophagectomy

^a^Fisher’s exact test

### EORTC QLQ-C30

Table 3 shows the transmediastinal esophagectomy and the transthoracic esophagectomy groups’ postoperative QOL scores measured using EORTC QLQ-C30. The global health status, physical, role, and cognitive function scale scores were significantly higher in the transmediastinal esophagectomy group than in the transthoracic esophagectomy group (*P* = 0.004, < 0.0001, 0.002, respectively). In the remaining functional scales, no significant differences were found. Fatigue, nausea and vomiting, pain, and appetite loss scores were significantly lower in the transmediastinal esophagectomy group than in the transthoracic esophagectomy group (*P* = 0.003, 0.032, 0.025, 0.018, respectively). The remaining symptom scales showed no significant differences.

**Table 3:** Postoperative QOL scores in NTTE and TTE using EORTC QLQ-C30

	NTTE (*n* = 26)	TTE (*n* = 37)	*P* value^a^
	Median (range)		
Global health status	83.3 (33.3–100)	66.7 (8.3–100)	0.004^a^
Functional scales			
Physical	100 (73.3–100)	86.7 (20–100)	< 0.0001^a^
Role	100 (10–100)	83.3 (0–100)	0.007^a^
Emotional	91.7 (33.3–100)	75 (0–100)	0.113
Cognitive	100 (50–100)	66.7 (16.7–100)	0.002^a^
Social	100 (33.3–100)	83.3 (16.7–100)	0.090
Symptom scales			
Fatigue	22.2 (0–55.6)	44.4 (0–100)	0.003^a^
Nausea and vomiting	0 (0–33.3)	0 (0–100)	0.032^a^
Pain	0 (0–33.3)	16.7 (0–100)	0.025^a^
Dyspnea	33.3 (0–33.3)	33.3 (0–100)	0.655
Insomnia	0 (0–66.7)	33.3 (0–100)	0.294
Appetite loss	0 (0–66.7)	33.3 (0–100)	0.018^a^
Constipation	0 (0–100)	0 (0–100)	0.317
Diarrhea	0 (0–100)	0 (0–100)	0.652
Financial difficulties	0 (0–33.3)	0 (0–100)	0.336

*NTTE* nontransthoracic esophagectomy, *TTE* transthoracic esophagectomy

^a^Wilcoxon rank-sum test

### EORTC QLQ-OES18

Table 4 shows the same two groups’ postoperative QOL scores measured using EORTC QLQ-OES18. The reflux and taste scores were significantly lower in the transmediastinal esophagectomy group than in the transthoracic esophagectomy group (*P* = 0.001, 0.041, respectively). The remaining scales showed no significant differences.

**Table 4:** Postoperative QOL scores between NTTE and TTE using EORTC QLQ-OES18

	NTTE (*n* = 26)	TTE (*n* = 37)	*P* value^a^
	Median (range)		
Symptom scales			
Eating	16.7 (0–58.3)	25 (0–91.7)	0.108
Reflux	0 (0–33.3)	16.7 (0–66.7)	0.001^a^
Pain	0 (0–55.6)	11.1 (0–66.7)	0.241
Trouble swallowing saliva	0 (0–33.3)	0 (0–33.3)	0.092
Choking when swallowing	0 (0–66.7)	0 (0–100)	0.687
Dry mouth	0 (0–66.7)	0 (0–100)	0.544
Trouble with taste	0 (0–66.7)	0 (0–100)	0.041^a^
Trouble with coughing	0 (0–66.7)	0 (0–66.7)	0.093
Trouble talking	0 (0–33.3)	0 (0–66.7)	0.456
Functional scales			
Dysphagia	88.9 (33.9–100)	88.9 (11.1–100)	0.988

*NTTE* nontransthoracic esophagectomy, *TTE* transthoracic esophagectomy

^a^Wilcoxon rank-sum test

## Discussion

We performed transmediastinal esophagectomy using a robotic surgical system to reduce postoperative pulmonary complications after esophagectomy [7]. Our findings suggest that this produces an improvement in the postoperative course and noninferiority in the pathological outcome, but its superiority in the long-term outcome was not investigated in our previous study. To evaluate surgical patients’ postoperative QOL, comparisons have been made between video-assisted thoracoscopic esophagectomy and open transthoracic esophagectomy and between transhiatal esophagectomy and transthoracic esophagectomy. Video-assisted thoracoscopic esophagectomy was found to be superior to open transthoracic esophagectomy in terms of global health status, physical function, fatigue, pain, and dyspnea [14], while transhiatal esophagectomy was superior to open transthoracic esophagectomy in terms of global health status, reflux, and odynophagia [15].

The superiority of QOL in conventional transhiatal esophagectomy may be explained by the reduced lymph dissection along the vagal nerve system; however, the effect on patients’ QOL after transmediastinal esophagectomy accompanying radical mediastinal dissection is still unknown. Dissecting the middle mediastinal lymph nodes such as the parabronchial or paracarinal nodes injures the pulmonary branches of the vagus [16] and such injuries may have negative effects on patients’ QOL. As for the recurrent laryngeal nerve, radical lymph dissections along the nerve may impair the patients’ QOL. The present study aimed to evaluate, using the EORTC questionnaires, the benefit of a robot-assisted radical transmediastinal procedure in terms of postoperative QOL compared to the transthoracic procedure.

Generally, transmediastinal esophagectomy is associated with better QOL scores in many measures. Above all, the reflux scores in QLQ-OES18 have been found to be superior in the transmediastinal esophagectomy group while several studies have found no significant difference in postoperative reflux scores in comparisons between video-assisted thoracoscopic esophagectomy and an open approach [14, 17]. Nonetheless, this finding is concordant with the previous report demonstrating better reflux scores in transhiatal esophagectomy patients than in transthoracic esophagectomy patients [15]. Although there are significant differences in the anastomotic sites—the frequency of the cervical as opposed to the intrathoracic anastomosis being significantly higher in the transmediastinal esophagectomy group than in the transthoracic esophagectomy group—the impact of the anastomotic site on postoperative QOL remains controversial [17–19]. Therefore, transmediastinal esophagectomy’s superior reflux scores might be explained not only by the anastomotic site but also by the surgical approach itself.

There are many limitations in our study: there was no assessment of preoperative QOL, and the cross-sectional study design did not enable us to investigate changes in QOL scores within individuals over time. To confirm the superiority of QOL in patients undergoing transmediastinal radical esophagectomy, another prospective study that includes survival analyses with adequate follow-up periods should be conducted. Furthermore, the transthoracic group had a higher proportion of exclusions from this study due to mortality. Although there was no significant difference in the pathological stage between two groups, this difference in mortality might be attributable to a higher frequency of advanced-stage disease in the transthoracic group, which had significantly higher primary tumor status and more (though not significantly) pathological stage III cases. This study can also be criticized for lacking any comparisons with minimally invasive esophagectomy. The improved QOL scores in the transmediastinal group of our current study should be interpreted as being significantly affected by the invasiveness of the transthoracic procedure. Among the QOL scales in the EORTC QLQ-C30 and QLQ-OES18, global health status, the functional scales (physical, role, and cognitive), and the symptom scales (fatigue, nausea and vomiting, pain, appetite loss) in QLQ-C30 might reflect differences between the invasiveness of the approaches, while symptom scales in QLQ-OES18 (reflux and taste) might reflect the better QOL of transmediastinal esophagectomy, as mentioned previously.

In conclusion, this cross-sectional study indicates that robot-assisted radical transmediastinal esophagectomy is associated with better QOL compared to transthoracic esophagectomy. Larger, prospective analyses are needed to confirm these results.
